# A Marek’s Disease Virus Messenger RNA-Based Vaccine Modulates Local and Systemic Immune Responses in Chickens

**DOI:** 10.3390/v16071156

**Published:** 2024-07-18

**Authors:** Fatemeh Fazel, Ayumi Matsuyama-Kato, Mohammadali Alizadeh, Jiayu Zheng, Charlotte Fletcher, Bhavya Gupta, Myles St-Denis, Nitish Boodhoo, Shayan Sharif

**Affiliations:** Department of Pathobiology, Ontario Veterinary College, University of Guelph, Guelph, ON N1G 2W1, Canada

**Keywords:** Marek’s disease virus, mRNA vaccine, immune response, cytokines, chicken

## Abstract

Marek’s disease (MD), caused by the Marek’s disease virus, is a lymphoproliferative disease in chickens that can be controlled by vaccination. However, the current vaccines can limit tumor growth and death but not virus replication and transmission. The present study aimed to evaluate host responses following intramuscular injection of an mRNA vaccine encoding gB and pp38 proteins of the MDV within the first 36 h. The vaccine was injected in low and high doses using prime and prime-boost strategies. The expression of type I and II interferons (IFNs), a panel of interferon-stimulated genes, and two key antiviral cytokines, IL-1β and IL-2, were measured in spleen and lungs after vaccination. The transcriptional analysis of the above genes showed significant increases in the expression of MDA5, Myd88, IFN-α, IFN-β, IFN-γ, IRF7, OAS, Mx1, and IL-2 in both the spleen and lungs within the first 36 h of immunization. Secondary immunization increased expression of all the above genes in the lungs. In contrast, only IFN-γ, MDA5, MyD88, Mx1, and OAS showed significant upregulation in the spleen after the secondary immunization. This study shows that two doses of the MDV mRNA vaccine encoding gB and pp38 antigens activate innate and adaptive responses and induce an antiviral state in chickens.

## 1. Introduction

Marek’s disease virus (MDV) is an oncogenic and highly contagious alpha herpesvirus in chickens [1]. This virus is considered a major global challenge in the poultry industry and is estimated to cause annual losses of approximately USD 1–2 billion [2].

Protective vaccines against Marek’s disease (MD) have been widely used since 1969 [3]. Due to the evolution of viral strains selecting for higher virulence, the need to develop vaccines with heightened protection has increased. Hence, polyvalent and Rispens vaccines were introduced due to their enhanced efficacy [4,5]. Although MD vaccines protect against tumorigenesis, death, and can ease economic burden, they do not prevent virus replication and shedding from infected chickens. After vaccination, continuous virus shedding still poses a risk to non-vaccinated chickens, which might lead to the emergence of more virulent pathotypes. Ideally, a successful vaccine is one that decreases or blocks viral replication and shedding.

The MDV genome encodes several glycoproteins. Glycoprotein (g)B is one of the main surface glycoproteins of MDV and aids in viral attachment to host cells by forming a heterodimer with other surface glycoproteins [6]. Another MDV protein, phosphoprotein 38 (pp38), is one of the early proteins expressed in the cytolytic infection of B and T cells and is involved in maintaining the transformed state of the infected cell. Accordingly, pp38 is also expressed in tumor cells [7]. The antigenic potential of both gB and pp38 to initiate cytotoxic T-cell immune responses has been previously characterized [6,8]. Boodhoo et al. have also reported a direct association between the magnitude of the T-cell immune response to pp38 antigen and host resistance to MDV [9].

In recent years, mRNA vaccines have been shown to trigger immune responses against various viruses in different animal species [10,11,12]. Nelson et al. compared an mRNA vaccine expressing full-length gB with vaccines expressing subunit proteins in New Zealand White rabbits infected with human cytomegalovirus [13]. The immune response elicited by the mRNA vaccine demonstrated the superior durability of the antibody response and had a greater breadth of peptide-binding responses [13]. Another mRNA vaccine encoding gE2, gD2, and gC2 of herpes simplex virus 2 (HSV-2) induced potent CD4^+^ T-follicular helper cell and germinal center B cell responses in mice [14]. In a different study, an 80–100% reduction in virus vaginal shedding was observed after mRNA vaccination against the surface glycoproteins of herpes simplex virus 1 (HSV-1) in mice [15]. Various studies have also reported on the efficacy and safety of the mRNA vaccine in chickens. Xu et al. (2023) created an mRNA vaccine that encodes the hemagglutinin (HA) protein from avian influenza virus (AIV), and subsequently evaluated its safety and protection. The administration of the mRNA-lipid nanoparticle (LNP) induced a significant increase in the expression of interferon-γ (IFN-γ) and reduced viral loads in various organs when compared with the control group [16]. In another study by Irshad et al. (2020), an AIV mRNA vaccine packaged in protein-coated chitosan nanoparticles (CNPs) was assessed. Following two immunizations, and subsequent AIV challenge, chickens in the mRNA vaccine groups showed lower viral load, reduced virus-originated pathology, and stronger immune responses compared to the control group. Significant CD4^+^ T-cell responses against the vaccine antigen were highlighted in the study [11].

Recently, our team has reported efficacy, tolerability, and protection induced by an mRNA vaccine encoding gB and pp38 proteins of MDV in chicken [17]. The present study explored the immune response induced by our mRNA-constructed vaccine and the mechanisms that may be involved in protecting against MDV using a prime and prime-boost vaccination strategy [18].

The immunostimulatory effects following two doses of mRNA vaccine and gB and pp38 expression were examined. The spleen (a major secondary lymphoid organ in chickens), and the lungs (an indicator organ of vaccine-induced mucosal immunity) were chosen to evaluate the expression of genes associated with anti-viral immune responses. In addition, the stability of the mRNA vaccine at the site of injection in the first 36 h of administration was assessed.

## 2. Materials and Methods

### 2.1. Plasmids and Cloning

The expression cassettes encoding the gB and pp38 antigens were manufactured by Thermo Fisher Scientific gene art synthesis (Regensburg, Germany). *Eco*RI-HF (NEB, Mississauga, ON, Canada) and *Kpn*I-HF (NEB, Canada) were used for plasmid linearization. The target genes were then loaded into PSF-T7- T7 PROMOTER PLASMID (Sigma-Aldrich, Steinheim, Germany). DH5α competent cells were used to generate a sufficient amount of PSF-T7-gB and PSF-T7-pp38 recombinant plasmids. The plasmids were isolated using a plasmid kit (Qiagen, Hilden, Germany).

The DNA constructs consisted of the following parts: the coding sequence of full-length MDV RB1B gB or pp38 antigens flanked by untranslated regions (UTRs), poly (A) at the 3′ end, Kozak consensus sequence (GCCACC), and a V5 tag (GTCATCCGGATAGGGATTGGGAGAGGAGCCAGAGCTAAGATG). An endosomal targeting motif of the chicken invariant chain (Ch-Ii; accession number: AY597053) was incorporated in the N-terminal of the antigen sequence.

### 2.2. mRNA Synthesis and Purification

Hi-T7 RNA polymerase (NEB, Canada) was used for in vitro mRNA transcription. Hi-T7 RNA polymerase, 10× Hi-T7 RNA polymerase reaction buffer, 1 μg of gB/pp38 template DNA, and ribonucleotide solution mix (NEB, Canada), were incubated at 50 °C for one hour in a thermocycler (Biometra, Göttingen, Germany). Trizol (Thermo Fisher Scientific, Mississauga, ON, Canada) was used for mRNA purification according to a previously described protocol [19]. Purified RNA was subsequently treated with a DNase enzyme (Ambion, Carlsbad, CA, USA). The concentration of purified mRNA was measured using the Nanodrop spectrophotometer (Thermo Fisher Scientific, Canada). The 5′ end of mRNA was capped using an enzymatic capping system that uses the vaccinia virus capping enzyme (NEB, Canada). Briefly, 1 μg of RNA was diluted in 15 μL of nuclease-free H_2_O. The RNA samples were then heated at 65 °C for 5 min, followed by cooling on ice for 5 min. The following ingredients were then added to the original RNA tubes: 1 μL of 10 mM guanosine-5′-triphosphate (GTP), 2 μL of 10× capping buffer, 1 μL of 2 mM S-adenosylmethionine (SAM), and 1 μL of Vaccina capping enzyme. Tubes were incubated at 37 °C for 30 min and were frozen at −80 °C.

### 2.3. Lipid Nanoparticle Preparation and Characterization

Lipid nanoparticles were formulated as previously described with minor modifications [20]. Lyophilized lipids, including ionizable lipid (MedKoo Biosciences, Durham, NC, USA), DSPC (Avanti Polar, Alabaster, AL, USA), cholesterol (Sigma-Aldrich, BSt. Louis, MO, USA), and PEG-lipid (Avanti Polar, USA), were dissolved in ethanol at a molar ratio of 50:10:38.5:1.5, respectively (ionizable lipid/DSPC/cholesterol/PEG-lipid). The in vitro transcribed gB/pp38 mRNA was added to 50 mM acidic citrate buffer, pH 4.0 (Teknova, Hollister, CA, USA). Purified gB/pp38 mRNA was then added to the lipid mixture. The nitrogen groups (N) to phosphate groups (P) (N:P) ratio was 3, and the ratio of aqueous to ethanol was also 3:1. Following a gentle pipetting, an ultrasonic processor (Thermo Fisher Scientific, Waltham, MA, USA) at 60% pulse for 90 s (10 s ON and 5 s OFF) was used to further mix the lipid–mRNA mixture. The test tubes containing lipid mix and mRNA were placed on ice during sonication to cool down the mixture. A dialysis cassette (Fisher Scientific, USA) was used to subsequently dialyze the product at 4 °C for at least 18 h buffer exchange. All mRNA:LNP packaging was completed the day before administration of the vaccine, and the mRNA:LNP mixture was used immediately after dialysis.

Zetasizer Nano ZS (Malvern Instruments Ltd., Cambridge, UK) was used to assess the physicochemical characteristics of LNPs such as particle size (dynamic light scattering (DLS)) and surface charge. Ribogreen dye (Thermo Fisher Scientific, USA) was also used to measure the encapsulation efficiency.

### 2.4. Cell Culture

In vitro stability of the gB/pp38 mRNA was assessed using the DF-1 chicken fibroblast cell line (obtained from Dr. Andrew Bendall, University of Guelph, Guelph, ON, Canada). DF-1 cells were maintained in EMEM complete medium (10% FBS + 1% Penicillin-Streptomycin (Thermo Fisher Scientific, Canada)). Five hundred thousand cells were seeded per well in a six-well plate in EMEM complete medium (10% FBS + 1% Penicillin-Streptomycin). When 80% confluent, cells were transfected with 1 μg mRNA encoding gB and pp38 using Lipofectin™ Transfection Reagent (Invitrogen, Carlsbad, CA, USA) for 4 h in optimum media (Thermo Fisher Scientific, USA). At 6-, 12- and 32-h post-transfection, cells were washed with PBS, treated with trypsin, and collected in Trizol for RNA extraction.

### 2.5. Experimental Animals

One-day-old specific-pathogen-free (SPF) White Leghorn chickens were received from the Canadian Food Inspection Agency (CFIA, Ottawa, ON, Canada). The chicks were randomly grouped in Horsfall units and were sheltered in the Animal Isolation Unit at the University of Guelph. Chickens had ad libitum access to food and water over the period of the experiment. The protocols used in this research project were reviewed and approved by the University of Guelph Animal Care Committee (Animal Utilization Protocol #4328).

### 2.6. Experimental Design

One-hundred-and-twenty SPF chicks were delivered to the Animal Isolation Unit and divided into three groups (*n* = 30 chicks/group). The next day, all chicks were treated as follows: Group 1: 200 μL of diluent control (PBS/chick); Group 2: intramuscular (IM) inoculation (iliotibialis muscle) with 2 μg gB + 2 μg pp38 per chick of mRNA packaged in LNPs (low-dose group); and Group 3: IM inoculation (iliotibialis muscle) with 4 μg gB + 4 μg pp38 per chick of mRNA packaged in LNPs (high-dose group). The vaccinated groups (Group 2 and Group 3) were boosted 14 days later. The control birds (Group 1) were injected with 200 μL/chick of PBS in lieu of the booster vaccination. Birds were observed three times a day at a minimum throughout the entirety of the study (including immediately following vaccination) to ensure no gross adverse effects. On 6, 12, 24, and 36 h post-prime and boost injection, six birds from each group were euthanized by CO_2_ inhalation for spleen, lung, and muscle sample collection. Muscle tissue was sampled only from prime groups. All samples were stored in RNAlater (Thermo Fisher Scientific, Vilnius, Lithuania) at 4 °C for 24 h and at −20 °C until RNA extraction.

### 2.7. RNA Extraction and cDNA Synthesis

Trizol reagent (Thermo Fisher Scientific, Mississauga, ON, Canada) was utilized for RNA extraction as previously described [21]. Briefly, one cm^2^ tissue samples were homogenized in 1 mL of Trizol, and DF1 cells and HEK 293 T cells were incubated for 2 min in Trizol, then mixed with chloroform (Sigma-Aldrich, USA). Isopropanol and 75% ethanol were used to precipitate and wash the extracted RNA. Pellets were resuspended in ultra-pure distilled water (Invitrogen, Grand Island, NY, USA). Extracted RNA was treated with DNase enzyme (Ambion, USA), and cDNA synthesis was performed using Superscript II (Life Technologies, Carlsbad, CA, USA). To measure the quantity and quality of the extracted RNA, Nanodrop spectrophotometry at 260 and 280 nm wavelength (Thermo Fisher Scientific, USA) was utilized.

### 2.8. Real-Time PCR

Quantitative real-time polymerase chain reaction was performed using SYBR green dye and the LightCycler 480 II (Roche Diagnostics, Laval, QC, Canada). Briefly, the plate was pre-incubated at 95 °C for 5 min followed by 40 to 50 cycles of 95 °C for 20 s, and 58 °C–64 °C (primer-specific annealing temperatures) for 15 s, in addition to 10 s elongations at 72 °C. The melt curve was generated by a 10 s incubation at 95 °C. The reaction then cooled down to 65 °C for 1 min, followed by heating to 95 °C. All primers were synthesized by Sigma-Aldrich (Oakville, ON, Canada) and listed in Table 1.

β-actin was used as a housekeeping gene for all relative gene expression analysis. Relative expression was calculated using the LightCycler© 480 software (Roche Diagnostics, Singapore). The relative expression of 5–6 replicates was compared to the PBS control group ± standard error.

### 2.9. Statistical Analysis

Gene expression data were analyzed with the Kruskal–Wallis test followed by the Mann–Whitney test. *p* ≤ 0.05 (*) was considered to be statistically significant. Statistical analysis was performed using GraphPad Prism version 9 (GraphPad Software, La Jolla, CA, USA).

## 3. Results

The physicochemical characterization of lipid nanoparticles and protein expression of this vaccine have been reported previously [17].

### 3.1. Detection of mRNA Molecules Encoding gB and pp38 in DF1 Cells

**Figure 1:**
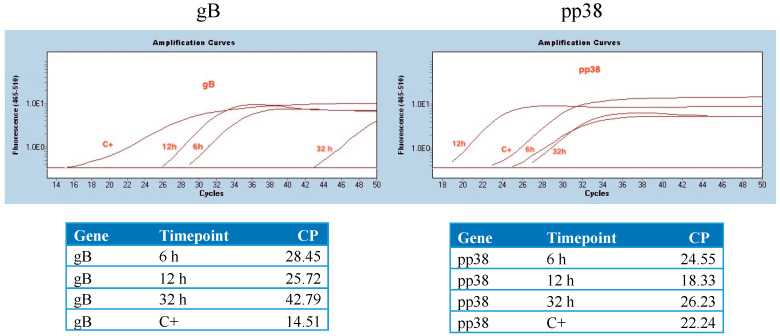
**In vitro stability of mRNA vaccine**. In vitro stability of gB-RB1B (**left**) and pp38-RB1B (**right**) mRNA molecules was analyzed within 6 to 32 h post-transfection in DF-1 cells. Five hundred thousand cells were seeded per well in a six-well plate in EMEM complete medium (10% FBS+ 1% pen-strep). Cells were transfected with 1 μg mRNA encoding gB and pp38 using Lipofectin for 4 h in the optimum medium (cells in the C+ group were infected with MDV). At 6-, 12- and 32 h post-transfection, cells were washed with PBS and collected following trypsin treatment for RNA extraction. Expression of gB and pp38 was analyzed by real-time PCR.

DF-1 cells were transfected with 1 μg of in vitro transcribed mRNA (gB, pp38) at three time points (6 h, 12 h, 32 h). At 12 h, the absolute quantity of both gB and pp38 was higher than those at 6 h and 32 h (Figure 1).

### 3.2. Detection of mRNA Molecules Encoding gB and pp38 at the Site of Injection

**Figure 2:**
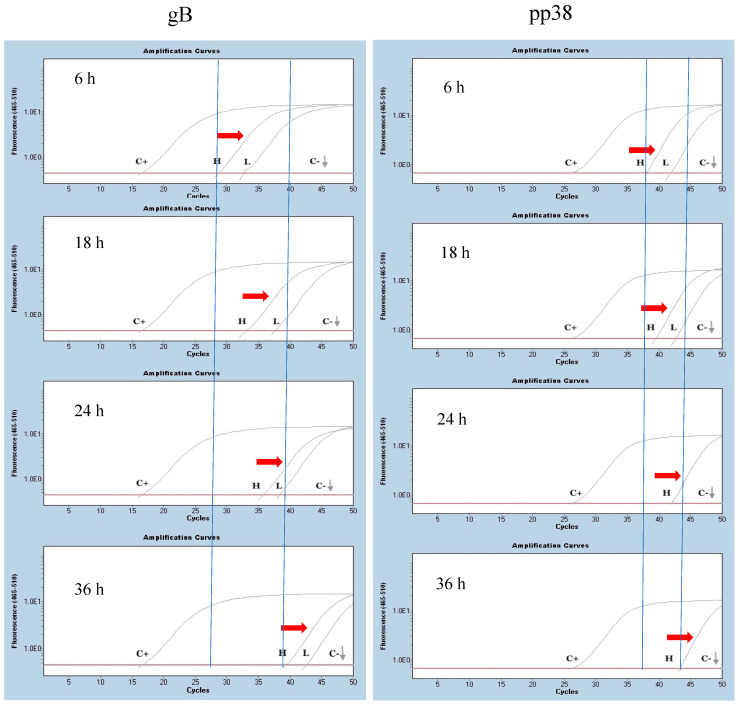
**In vivo stability of mRNA vaccine.** In vivo stability of gB-RB1B (**left** panel) and pp38-RB1B (**right** panel) mRNA molecules was analyzed within 6 to 36 h post-IM administration at the injection site. One cm^2^ muscle sample from the injection site (iliotibial muscle) was excised and processed for RNA extraction and cDNA synthesis at six-, 18-, 24-, and 36-h post-IM administration of PBS (C−, low-dose mRNA (L). and high-dose mRNA (H). Positive control (C+) is DNA extracted from a lung tissue sample from an infected chicken with RB1B MDV at 21 dpi. Red arrows show a decrease in the absolute amount of injected mRNA at each time point. Gray arrows point to the ribogreen emission of the control negative (C−) which is baseline.

The in vivo stability of mRNA molecules encoding gB and pp38 proteins was analyzed at the injection site (iliotibial muscle) at 6, 12, 24, and 36 h post-administration (hpa). At each time-point, the absolute amount of mRNA showed a 3–4 log reduction for gB and a 2.5 log reduction for pp38 compared to the initial measurement at the first time-point (six hpa) in the both the low-dose and high-dose groups. In the low-dose group, the pp38 mRNA was not detectable at 24 hpa and 36 hpa time points (Figure 2).

### 3.3. Expression of Cytokine Genes and Interferon-Stimulated Genes (ISGs) after Administration of mRNA Molecules Encoding MDV gB and pp38 Proteins

#### 3.3.1. Spleen

To analyze mRNA-driven changes following mRNA vaccination in chickens, tissue samples were collected at 6, 18, 24, and 36 h post-vaccine administration, and the relative expression of a panel of interferons, interferon regulatory genes, and two cytokines (interleukin (IL)-β and IL-2) was measured.

**Figure 3:**
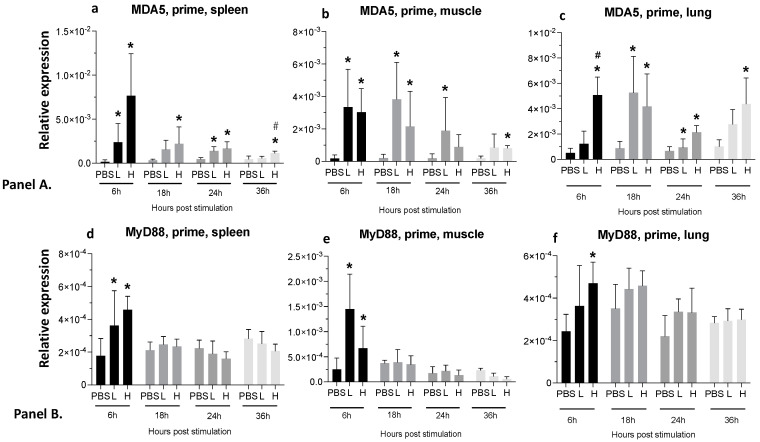
**Relative expression of MDA5 and MyD88 genes at 6-, 18-, 24-, and 36-h post-vaccine administration in muscle, spleen, and lungs.** (Panel **A**). Relative expression of MDA5 in the spleen (**a**), muscle (**b**), and lungs (**c**). (Panel **B**). Relative expression of MyD88 in the spleen (**d**), muscle (**e**), and lungs (**f**) post-IM administration of low-dose mRNA (L), high-dose mRNA (H), and PBS control. Relative expression data represent the mean fold-change of 5–6 biological replicates (chickens) compared to the PBS-treated control group (*) and low-dose group (#) ± standard error. Data were analyzed with the Kruskal–Wallis test followed by the Mann–Whitney test (*p* ≤ 0.05 was considered statistically significant). ß-actin was used as a reference gene for all relative expressions.

Increased expression of melanoma differentiation-associated protein 5 (MDA5) and myeloid differentiation primary response 88 (MyD88) was detected in the spleen, lungs, and injection site of the birds six hours after receiving either a low- or high-dose of the prime vaccine. MDA5 expression remained significantly high until 36 h post-stimulation in the spleen and lungs of the high-dose group (Figure 3a,c). The high-dose group also showed significantly elevated MyD88 expression in the site of injection, spleen, and lungs at 6 hpa (Figure 3d–f).

**Figure 4:**
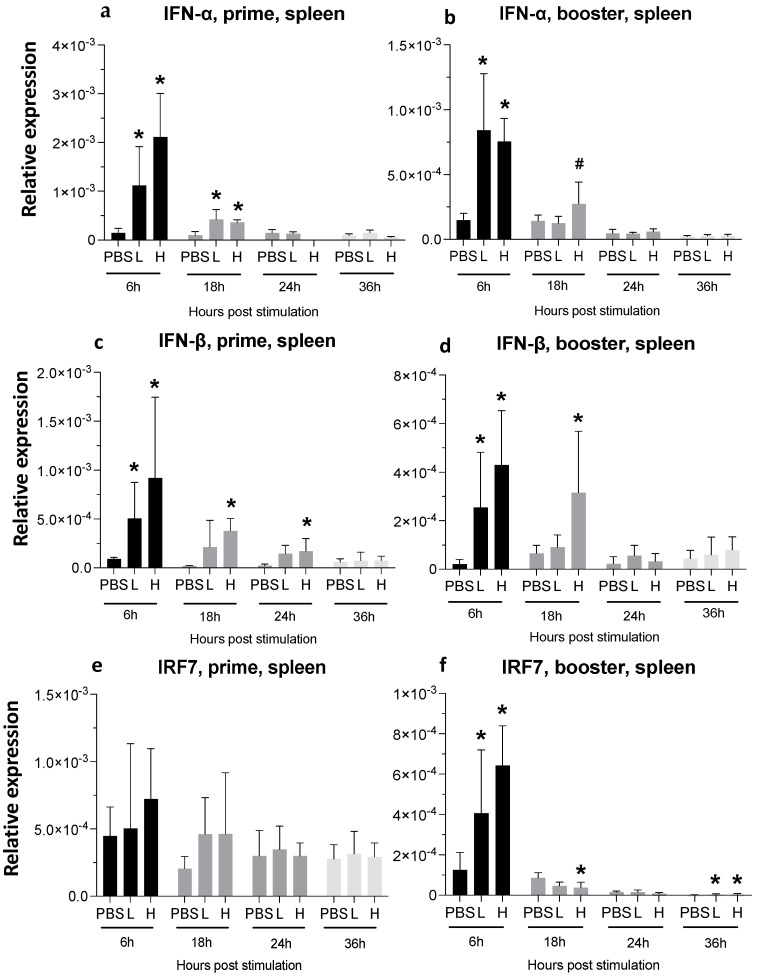
**Relative expression of IFN-α, IFN-β, and IRF7 in the spleen at 6-, 18-, 24-, and 36-h post-IM vaccine administration**. Graphs compare the relative expression of IFN-α (**a**,**b**), IFN-β (**c**,**d**), and IRF7 (**e**,**f**) in the spleen post-IM administration of low-dose mRNA (L), high-dose mRNA (H), and PBS control. Relative expression data represent the mean fold-change of 5–6 biological replicates (chickens) compared to the PBS-treated control group (*) and low-dose group (#) ± standard error. Data were analyzed with the Kruskal–Wallis test followed by the Mann–Whitney test (*p* ≤ 0.05 was considered statistically significant). ß-actin was used as a reference gene for all relative expressions.

Vaccination significantly increased the expression of IFN-α in both the low- and high-dose groups at 6 hpa. A single injection of a low-dose vaccine was able to significantly increase IFN-α expression in the spleen at 6 hpa, which decreased to one-half and one-third after 18 and 24 h of prime vaccination, respectively. The high dose of vaccine led to 4 times higher IFN-α expression at 6 hpa compared to at 18 hpa in the spleen (Figure 4a). IFN-α expression at the 6 h time-point following the booster dose was 2-fold higher than that at the 18 h time-point. Boosting the low-dose group led to almost 5-fold and 8-fold higher expression of IFN-α at 6 hpa compared to 18 h and 24 h time-points, respectively (Figure 4b).

The highest number of IFN-β transcripts was observed 6 h after prime vaccination, when it was 5-fold and 10-fold higher in the low- and high-dose groups compared to the PBS-treated controls, respectively. IFN-β expression remained significantly high until 18 hpa in the high-dose group after both prime and booster doses (Figure 4c,d).

One dose of vaccine did not alter the expression of interferon regulatory factors (IRF) 7 (Figure 4e); meanwhile, at 6 h after the booster injection, IRF7 expression had increased significantly (Figure 4f).

**Figure 5:**
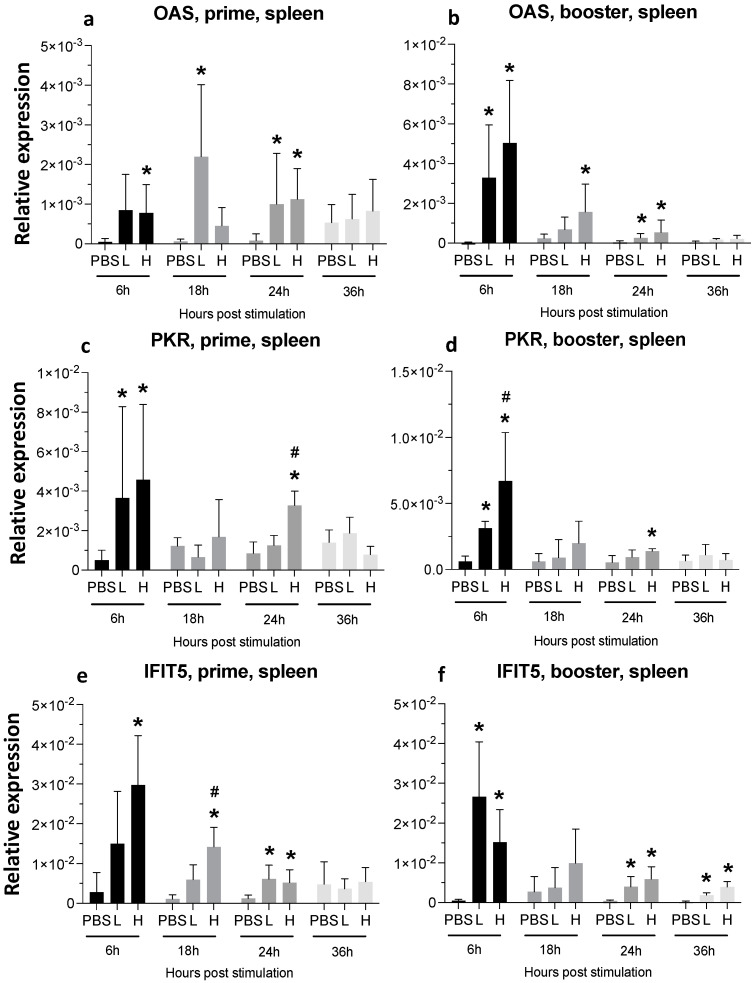
**Relative expression of OAS, PKR, and IFIT5 in the spleen at 6-, 18-, 24-, and 36-h post-IM vaccine administration in the spleen**. Graphs compare the relative expression of OAS (**a**,**b**), PKR (**c**,**d**), and IFIT5 (**e**,**f**) in the spleen post-IM administration of low-dose mRNA (L), high-dose mRNA (H), and PBS control. Relative expression data represent the mean fold-change of 5–6 biological replicates (chickens) compared to the PBS-treated control group (*) and low-dose group (#) ± standard error. Data were analyzed with the Kruskal–Wallis test followed by the Mann–Whitney test (*p* ≤ 0.05 was considered statistically significant). ß-actin was used as a reference gene for all relative expressions.

The expression of 2′,5′-oligoadenylatesynthase (OAS) peaked at 18 h following a single injection with low-dose mRNA and it remained elevated until 24 hpa (Figure 5a). Upon the booster administration, OAS expression increased early (2.5-fold and 4.5-fold higher in low-dose and high-dose groups, respectively, compared to the PBS-treated control group), and it remained significantly high until 24 hpa in the high-dose group (Figure 5b). PKR expression increased significantly by 6 h after the first and second injection in both the low- and high-dose groups (Figure 5c,d). The expression of dsRNA-dependent protein kinase (PKR) in the high-dose group was significantly higher than in the low-dose group at 6 h after booster injection (Figure 5d).

A high dose of the vaccine resulted in elevated expression of interferon-induced protein with tetratricopeptide repeats (IFIT) 5 that remained significantly high until 24 hpa in the prime group (Figure 5e). The booster injection also increased IFIT5 expression at 6, 24, and 36 hpa time-points in both the low- and high-dose groups (Figure 5f).

**Figure 6:**
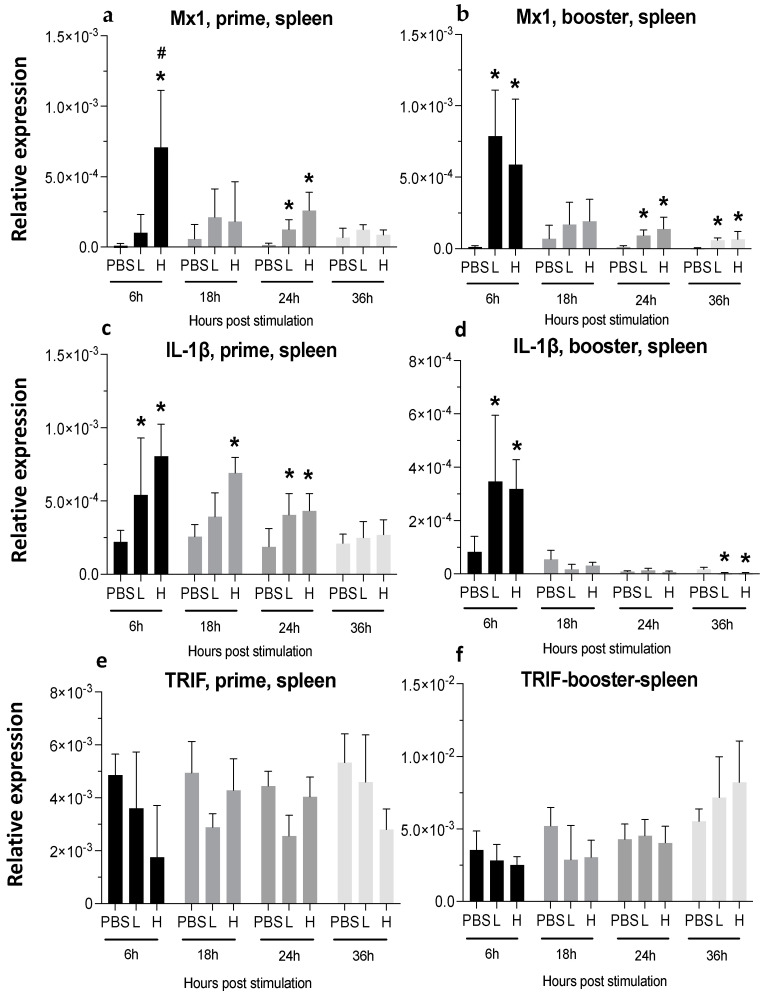
**Relative expression of Mx1, IL-1β, and TRIF at 6-, 18-, 24-, and 36-h post-IM vaccine administration in the spleen.** Graphs compare the relative expression of Mx1 (**a**,**b**), IL-1β (**c**,**d**), and TRIF (**e**,**f**) in the spleen post-IM administration of low-dose mRNA (L), high-dose mRNA (H), and PBS control. Relative expression data represent the mean fold-change of 5–6 biological replicates (chickens) compared to the PBS-treated control group (*) and low-dose group (#) ± standard error. Data were analyzed with the Kruskal–Wallis test followed by the Mann–Whitney test (*p* ≤ 0.05 was considered statistically significant). ß-actin was used as a reference gene for all relative expressions.

A single injection of low-dose mRNA did not significantly increase the Myxoma-resistance protein (Mx) 1 expression, whereas increasing the dose significantly elevated its expression at six hpa (Figure 6a). Following boosting, Mx1 expression was highest at 6 hpa and remained significantly high at the 24 and 36 hpa time-points in both the low-dose and high-dose groups (Figure 6b).

One dose of vaccine led to a 2-fold increase (low-dose) and a 3-fold increase (high-dose) in IL-1β expression, starting at 6 hpa and lasting until 24 hpa (Figure 6c). The second injection of mRNA also increased IL-1β expression by 3-fold at 6 hpa; this transient increase was no longer apparent at later time points (Figure 6d).

**Figure 7:**
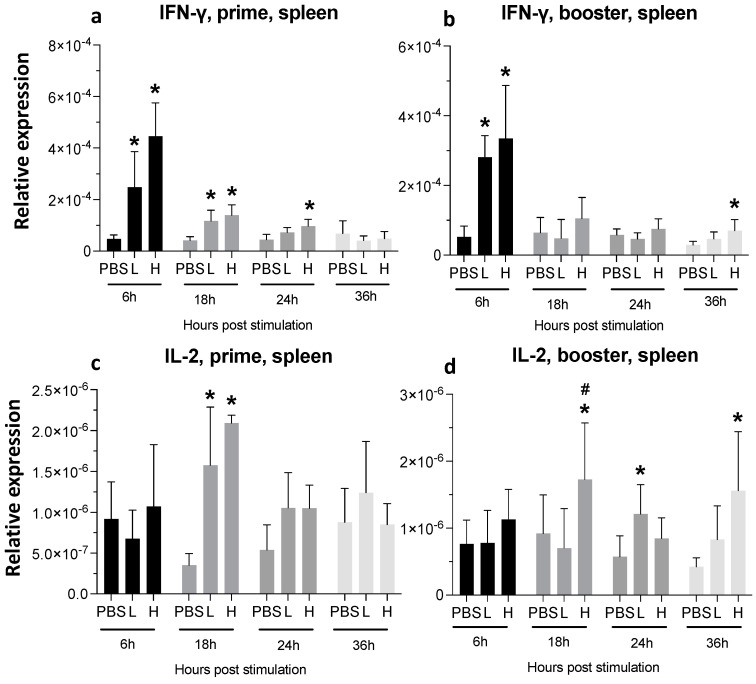
**Relative expression of IFN-γ and IL-2 at 6-, 18-, 24-, and 36-h post-IM vaccine administration in the spleen.** Graphs compare the relative expression of IFN-γ (**a**,**b**), and IL-2 (**c**,**d**) in the spleen post-IM administration of low-dose mRNA (L), high-dose mRNA (H), and PBS control. Relative expression data represent the mean fold-change of 5–6 biological replicates (chickens) compared to the PBS-treated control group (*) and low-dose group (#) ± standard error. Data were analyzed with the Kruskal–Wallis test followed by the Mann–Whitney test (*p* ≤ 0.05 was considered statistically significant). ß-actin was used as a reference gene for all relative expressions.

A single mRNA vaccine administration increased the expression of IFN-γ in both low- (10-fold increase) and high-dose (20-fold increase) at 6 hpa in the spleen. This expression decreased later but was still significantly higher until 24 hpa in the high-dose group (Figure 7a).

At 18 hpa, IL-2 expression was increased by 3-fold (low dose) and five times (high dose) (Figure 7c), and the second injection of high-dose mRNA resulted in a significantly increased expression of IL-2 at 18 hpa and 36 hpa (Figure 7d).

#### 3.3.2. Lungs

**Figure 8:**
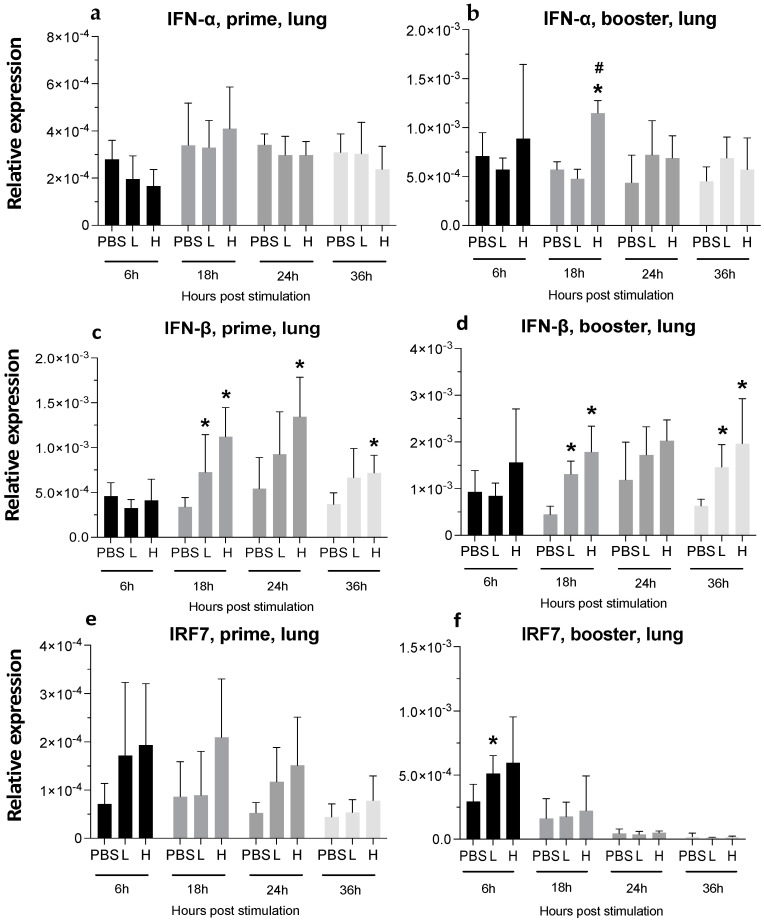
**Relative expression of IFN-α, IFN-β, and IRF7 in the lungs at 6-, 18-, 24-, and 36-h post-IM vaccine administration.** Graphs compare the relative expression of IFN-α (**a**,**b**), IFN-β (**c**,**d**), and IRF7 (**e**,**f**) in the lungs post-IM administration of low-dose mRNA (L), high-dose mRNA (H), and PBS control. Relative expression data represent the mean fold-change of 5–6 biological replicates (chickens) compared to the PBS-treated control group (*) and low-dose group (#) ± standard error. Data were analyzed with the Kruskal–Wallis followed by the Mann–Whitney test (*p* ≤ 0.05 was considered statistically significant). ß-actin was used as a reference gene for all relative expressions.

While a single dose of the mRNA vaccine did not alter IFN-α expression, boosting with the high dose of the vaccine led to an over 2-fold increase at 18 hpa (Figure 8a,b). A high dose of the vaccine also increased IFN-β expression by 2- and 3-fold after a single injection in the high-dose group at 18 hpa and 24 hpa, respectively. The elevated expression of IFN-β in the lungs was significant at 18 and 36 hpi after the booster injection (Figure 8c,d). One dose of the vaccine did not significantly alter IRF7 expression, but the booster dose increased the expression of IRF7, which was significant in the low-dose group at 6 hpa (Figure 8e,f).

**Figure 9:**
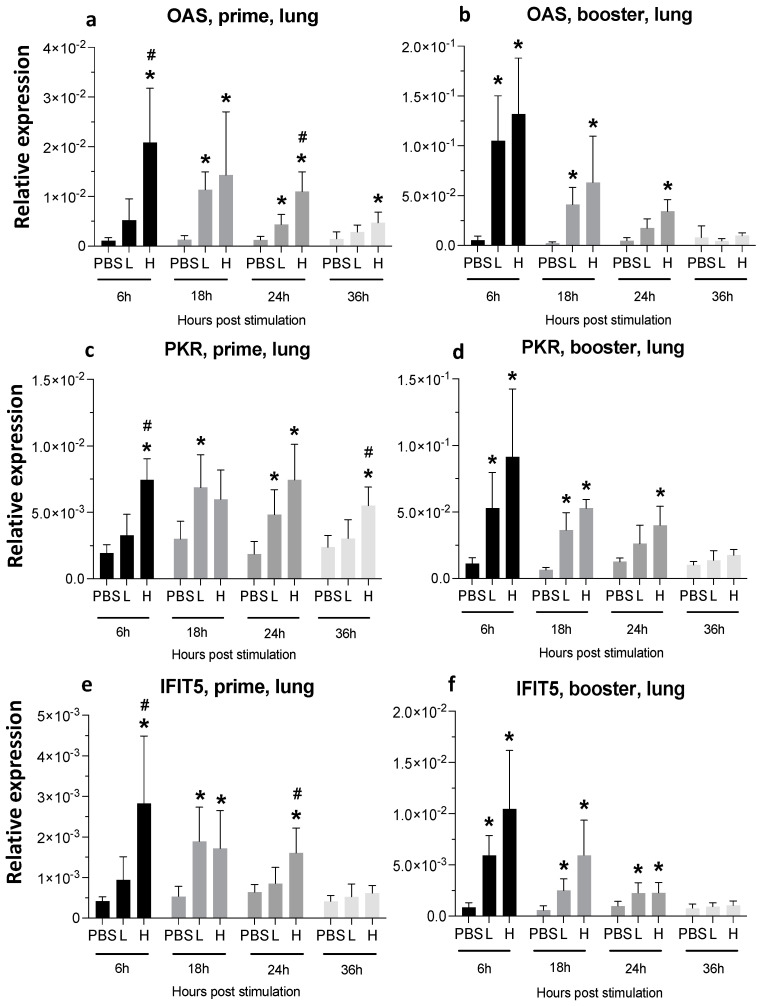
**Relative expression of OAS, PKR, and IFIT5 in the spleen at 6-, 18-, 24-, and 36-h post-IM vaccine administration in the lungs.** Graphs compare the relative expression of OAS (**a**,**b**), PKR (**c**,**d**), and IFIT5 (**e**,**f**) in the lungs post-IM administration of low-dose mRNA (L), high-dose mRNA (H), and PBS control. Relative expression data represent the mean fold-change of 5–6 biological replicates (chickens) compared to the PBS-treated control group (*) and low-dose group (#) ± standard error. Data were analyzed with the Kruskal–Wallis test followed by the Mann–Whitney test (*p* ≤ 0.05 was considered statistically significant). ß-actin was used as a reference gene for all relative expressions.

The single-dose and double-dose groups showed significantly higher OAS expression until 36 hpa and 24 hpa, respectively. At the earliest time-point (6 hpa), a 5-fold increase in the prime group and a 10-fold increase in the booster group were detected (Figure 9a,b). OAS expression in the high-dose group was significantly higher than the low-dose group at 6 and 24 h after prime dose injection.

PKR expression was elevated following a single high dose of the vaccine at 6, 24, and 36 hpa. After injection of the high-dose vaccine, PKR expression remained significantly high until 36 hpa in the booster groups (Figure 9c,d).

Following a high-dose injection in the prime group, IFIT5 expression increased by 5-fold, 4-fold, and 3-fold at 6 hpa, 18 hpa, and 24 hpa, respectively (Figure 9e). Boosting the chicks with another dose of mRNA led to a 10-fold increase at 6 hpa and a 5-fold increase at 18 hpa that dropped at 24 hpa but was still significantly higher when compared to PBS-treated controls (Figure 9f).

**Figure 10:**
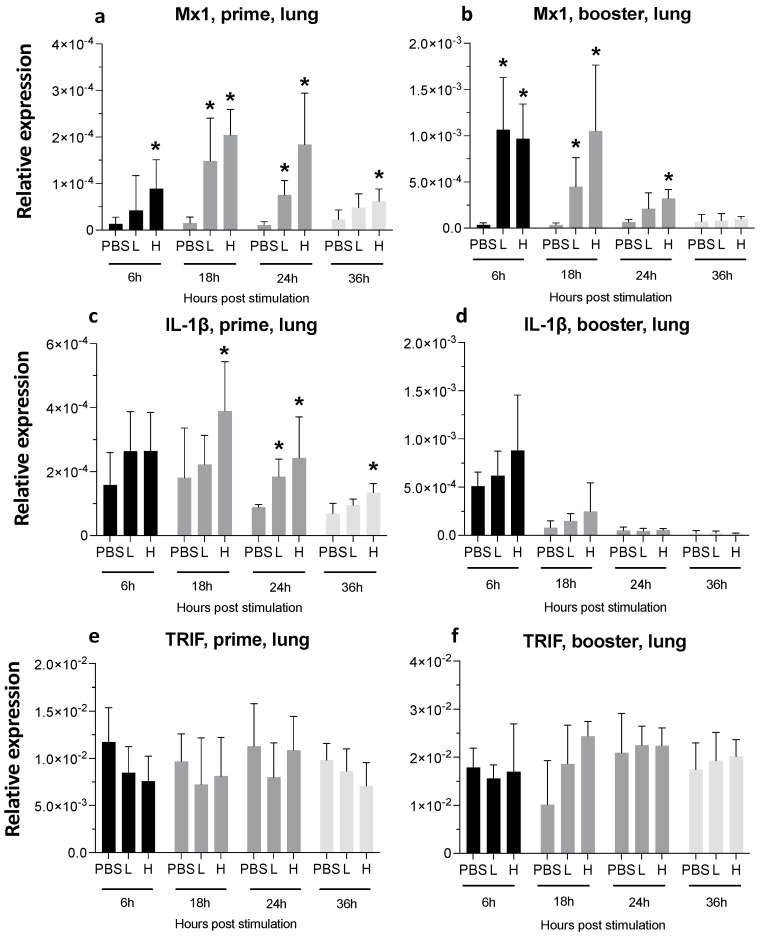
**Relative expression of Mx1, IL-1β, and TRIF at 6-, 18-, 24-, and 36-h post-IM vaccine administration in the lungs.** Graphs compare the relative expression of Mx1 (**a**,**b**), IL-1β (**c**,**d**), and TRIF (**e**,**f**) in the lungs post-IM administration of low-dose mRNA (L), high-dose mRNA (H), and PBS control. Relative expression data represent the mean fold-change of 5–6 biological replicates (chickens) compared to the PBS-treated control group (*) and low-dose group ± standard error. Data were analyzed with the Kruskal–Wallis test followed by the Mann–Whitney test (*p* ≤ 0.05 was considered statistically significant). ß-actin was used as a reference gene for all relative expressions.

Mx1 expression peaked at 18 h post-prime injection and stayed significantly high until 36 hpa in the high-dose group. The booster dose resulted in an earlier increase in Mx1 expression compared to the prime dose. Following the booster injection, Mx1 expression increased by around 20-fold at 6 hpa and 18 hpa. Elevated Mx1 expression had dropped to around 4-fold at 24 hpa in the booster group (Figure 10a,b). IL-1β expression was increased at 18 hpa in the prime group, and it stayed high until 36 hpa in the high-dose group (Figure 10c).

**Figure 11:**
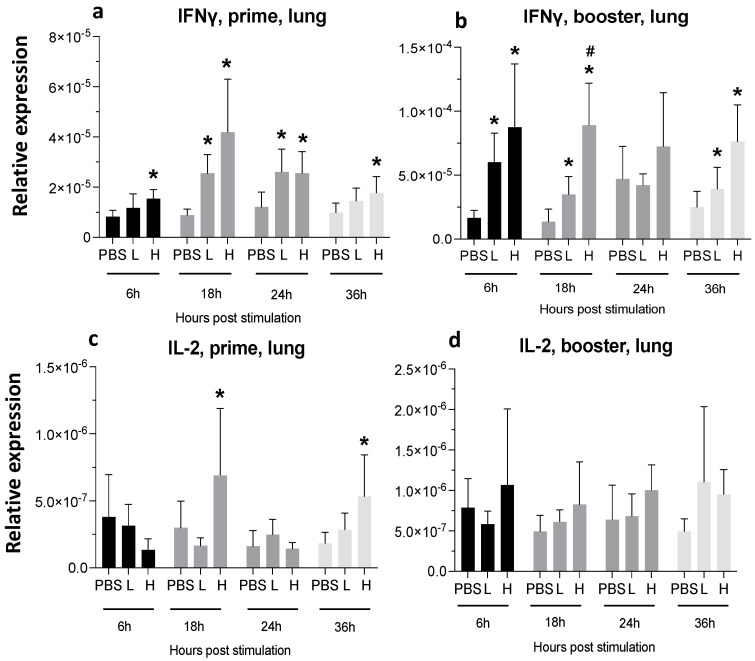
**Relative expression of IFN-γ and IL-2 at 6-, 18-, 24-, and 36-h post-IM vaccine administration in the lungs.** Graphs compare the relative expression of IFN-γ (**a**,**b**), and IL-2 (**c**,**d**) in the lungs post-IM administration of low-dose mRNA (L), high-dose mRNA (H), and PBS control. Relative expression data represent the mean fold-change of 5–6 biological replicates (chickens) compared to the PBS-treated control group (*) and low-dose group (#) ± standard error. Data were analyzed with the Kruskal–Wallis test followed by the Mann–Whitney test (*p* ≤ 0.05 was considered statistically significant).

IFN-γ expression in the lungs increased at 6 hpa and remained significantly high until 36 hpa after a single injection with the high-dose vaccine (Figure 11a). The second injection of mRNA resulted in a significant increase in IFN-γ expression in the lungs at 6, 18, and 36 hpa in both low- and high-dose groups (Figure 11b).

IL-2 expression in the lungs peaked at 18 hpa, and after a transient drop, it increased again at 36 hpa (Figure 11c). The booster injection did not cause a statistically significant increase in the IL-2 expression (Figure 11d).

Changes in the gene expression following booster injection were different in the spleen and lungs. Among the gene expression profiles analyzed in the spleen, only five genes (IFN-γ, MDA5, MyD88, Mx1, and OAS) showed significantly elevated expression after the booster dose compared to the same time point after the primary vaccination. In the lungs, all 13 genes showed a significant increase in all or at least one time-point after the booster dose (IFN-α, IFN-β, IFN-γ, MyD88, OAS, PKR, TRIF, and IL-2 genes showed upregulated expression in all four time-points).

## 4. Discussion

The in vitro stability of synthesized mRNA packaged in LNPs at four time points after injection was first assessed. At each time-point post-injection, the amount of mRNA molecules showed approximately a 3.5-log and 2.5-log reduction for gB and pp38 constructs, respectively. Although both gB and pp38 mRNAs were constructed using the same protocol, the size of the molecules and nucleotide composition may have impacted the speed of degradation in vivo. Higher G:C content within the genetic composition of the mRNA molecules can increase mRNA stability and its half-life inside the cell [29]. The G:C content of the mRNA encoding gB and pp38 proteins in this study was 42.7% and 51.9%, respectively. This may explain the slower degradation pace of pp38 compared to gB at the injection site (2.5 log decrease in each time-point for pp38 compared to 3.5 log decrease for gB). Although the major structures involved in mRNA stability, such as UTRs, 5′ cap, and polyA tail, were incorporated in the in vitro-synthesized mRNA molecules used in this study, we did not use modified nucleotides. By modifying the nucleotide composition of mRNA constructs, the half-life of mRNA in the cytosol increases, which will impact the efficiency of protein production from these constructs.

How mRNA distributes in the body upon injection is an important question that has been partially answered in other studies using different animal models. Generally speaking, the injection site, lymph nodes, and spleen are amongst the organs and tissues listed with the highest concentrations of the administered mRNA [30]. Bahl et al. (2017) compared the T_max_ (time of the maximum concentration) and T ½ (required time for the quantity of mRNA to be reduced to half) in different organs upon IM injection. The maximum concentration of mRNA in the muscle, spleen, and lung were recorded at two hpa. Injected mRNA dilutes to half its original amount in the muscle within 18 h, while the lung and spleen showed mRNA dilution at 16 and 25 h, respectively [30]. This agrees with the mRNA degradation time in the muscles observed in the present study.

To assess the immunostimulatory effects of the mRNA vaccine, we monitored the spleen and lungs for changes in the expression of type I and II interferons, a panel of ISGs, and two cytokines that are key players in antiviral response. Chickens lack draining lymph nodes, and their spleen is the most physiologically important secondary lymphoid organ. The lungs are the primary site of infection upon inhalation of MDV particles and represent the initial site of the host responses against the virus. Upon administration, mRNA is sensed by endosomal Toll-like receptors (TLRs) (TLR7 and 8) and cytoplasmic sensors (MDA5) [31]. TLRs bound to mRNA activates MyD88 and enables RNA recognition by MDA5, which consequently activates mitochondrial antiviral signaling protein (MAVS). Activation of these two adaptor proteins induces a signaling cascade resulting in the expression of genes encoding antiviral response elements, including type I IFNs [32]. MDA5 also plays a role in the phosphorylation of IRF7 through its caspase-recruitment domains (CARDs), resulting in increased expression of IFN-α and IFN-β [33].

The results obtained from the injection site, spleen, and lungs showed elevated expression of MDA5 and MyD88 at early vaccination time points. This agrees with the findings reported by Edwards et al. (2017) in an in vivo mouse model (C57BL/6 mouse) and in human cell lines in vitro [31]. Type I interferons can consequently induce ISGs after binding to their receptors. Mx, PKR, OAS, and IFIT5 are some of the ISGs highly associated with the antiviral response. Kato et al. transfected dendritic cells (DCs) with RNAs extracted from vesicular stomatitis virus and encephalomyocarditis virus and measured the production of IFN-α. Their results showed impaired production of IFN-α in MDA5^−/−^ mice, highlighting the role of MDA5 in sensing exogenous RNA and induction of IFN-α expression [33].

IL-1β is another pro-inflammatory cytokine produced following non-self RNA recognition by pattern recognition receptors (PRRs) in the cytosol. Increased IL-1β expression after mRNA administration in our study agrees with the results reported by others [31,34]. Upregulated expression of IL-1β can aid in the maturation of DCs resulting in increased antigen presentation and stronger adaptive immune responses after viral challenge [35]. In addition, IL-1 (mostly IL-1β) plays a major role in balancing the inflammatory response following mRNA injection. Tahtinen et al. (2022) showed that mice and humans reacted differently to equal relative doses of an mRNA vaccine. IL-1β expression following injection of two different ionizable lipids (MC3, SM-102) broadly used for LNPs formation was also analyzed [34]. It has been shown that empty LNPs made by MC3 lipids could not stimulate IL-1β production, while LNPs made by SM-102 (used in the COVID-19 vaccine designed by Moderna) were potent inducers of IL-1β [34]. As the LNPs in the present study were made with MC3, the elevated expression of IL-1β after vaccine administration is likely to be initiated by the mRNA molecules and not by the LNPs.

IFN-γ is the sole member of the type II IFN group and is secreted by activated T cells and natural killer (NK) cells. IFN-γ is considered a key player in the antiviral state, and its direct effect on the reduction of MDV has already been characterized [36]. The administration of recombinant chicken IFN-γ significantly boosted the immunity elicited by the turkey herpesvirus (HVT) vaccine against virulent MDV. As IFN-γ is mainly secreted by NK cells and activated T cells, elevated expression of IFN-γ in the lungs in this study suggests activation of Th1 CD4^+^ cells, CD8^+^ cytotoxic T cells, and NK cells following mRNA immunization of chickens.

IL-2 is a cytokine that is known to have a key role in T-cell activation, NK cell stimulation, and B cell expansion [37]. IL-2 reduces HSV-1 replication and pathogenicity in mice, as it was linked to the action of effector CD4^+^ and CD8^+^ T lymphocytes. Ghiasi et al. (2002) also observed increased virulence of HSV-1 after IL-2 depletion in mice [38] In chickens, IL-2 also plays a role in NK cell activation, lymphocyte proliferation, and defense against intracellular pathogens [39,40]. Reduced systemic viral load linked to increased expression of IL-2 during infection with a highly virulent strain of Newcastle disease virus in chickens is also reported [41]. To analyze possible T-cell activation following mRNA vaccination, we measured IL-2 expression in the spleen and lungs. Significantly elevated expression of IL-2 was demonstrated in both organs at 18 hpi following a single injection of the mRNA vaccine. IL-2 expression remained high until 36 hpi after booster injection in the spleen. Increased IL-2 expression was also reported following mRNA immunization against the Omicron variant of severe acute respiratory syndrome coronavirus 2 (SARS-CoV-2) [42]. Similarly, Jiang et al. measured IL-2 and IFN-γ expression in blood after immunization with a gD mRNA vaccine against pseudorabies virus and reported a 2-fold increase in IL-2 and IFN-γ expression in vaccinated mice [10].

A comparison of gene expression following immunization with low versus high doses of the vaccine showed that, for most genes and at most time points, the relative gene expression was higher in the high-dose group. However, these differences were only significant at one or two time-points for a select number of genes. This may be due to the activation of intracellular inhibitory mechanisms involved in the degradation and dilution of excessive non-self mRNA (such as exonucleolytic degradation) when the vaccine was injected at the higher dose. Determining the toxic and tolerable limits of non-self mRNA in chickens is an area that requires further investigation.

Changes in gene expression after the booster dose were monitored to assess the necessity of the booster injection. The spleen and lungs showed different patterns of gene expression after the second vaccination. Among the gene expression profiles analyzed in the spleen, only five genes (IFN-γ, MDA5, MyD88, Mx1, and OAS) showed significantly elevated expression after the secondary immunization when compared to the same time point after the first immunization. All 13 genes showed significant increases in all or at least one time-point in the lungs after the booster dose (IFN-α, IFN-β, IFN-γ, MyD88, OAS, PKR, TRIF, IL-2 genes showed upregulated expression in all four time-points). The reason different organs showed varied gene expression patterns after the second dose might be due to the diverse cell compositions in each organ following the primary immunization. The migration of various subsets of innate or adaptive immune system cells from the spleen to other organs (such as the lung, gastrointestinal system, etc.) might have affected transcriptional patterns in the spleens and lungs [43]. For instance, the expression of genes involved in effector cell activation, such as IL-2, was significantly increased at all time points in the lungs after the booster injection. This might have occurred due to the recruitment of activated NK cells and T cells to the lungs.

Nevertheless, mRNA cellular uptake may have differed in the spleen and lungs following the second injection. The cellular uptake of mRNA is affected by the number of extracellular proteins, such as apolipoprotein E (ApoE), which is responsible for lipid trafficking and facilitating LNP uptake [44]. Although the liver is the primary organ that synthesizes ApoE, it can also be secreted from cells within the lung itself. Alveolar macrophages are one such producer of ApoE in the lung, though they are known as free avian respiratory macrophages (FARMs) in birds [35,36]. Although the abundance of FARMs is much lower in birds compared to their mammalian counterparts, activated alveolar macrophages in the lungs following the first injection may have contributed to the higher ApoE secretion and higher LNP uptake in the lungs observed upon booster administration [45]. Further investigation is needed to determine if this is applicable to the present study. Mammalian studies characterized the pathways involved in ApoE secretion by bronchoalveolar macrophages and the consequently increased secretion of IL-1β by alveolar macrophages of the lungs [46]. Since ApoE levels were not measured in the present study, we speculate that the enhanced IL-1β expression in the high-dose group following the booster injection might be attributed to increased ApoE secretion by bronchoalveolar macrophages.

Our recent study has discussed vaccine-induced protection against MDV following two immunizations with high-dose mRNA, particularly focusing on its correlation with type I and II IFNs [17]. Significantly increased IFN-α expression was observed in the mRNA vaccine group at 21 days post-MDV infection compared to both the unvaccinated MDV-challenged and PBS-treated groups. Furthermore, mRNA-vaccinated groups exhibited elevated IFN-β expression compared to the PBS-treated group. Type I interferons have been shown to be downregulated by MDV [47]. Our MDV challenge trials revealed a negative correlation between MDV genome load and type I IFNs expression, likely due to lower MDV replication in the mRNA-vaccinated group (high-dose) and less subsequent inhibition of type I IFNs expression by the virus. Furthermore, we observed higher expression of type II interferon (IFN-γ) in chickens immunized with two high doses of the mRNA vaccine compared to the PBS-treated control group at 4 and 21 days post-infection (dpi). The crucial role of IFN-γ in the immune response against MDV has been previously discussed [9,48]. In our study, activated T cells were likely the main source of IFN-γ following two high-dose mRNA vaccination.

Taken together, our findings in the present study demonstrate that a bivalent mRNA vaccine encoding gB and pp38 proteins can induce early cytokine gene expression, resulting in elevated levels of IL-2 and IFN-γ. These cytokines are essential for effectively initiating adaptive immune responses against MDV.

To further investigate the antiviral response and anti-tumor function of this vaccine, additional in vivo and in vitro studies are needed. The antigen-specific T-cell response following viral challenge in vaccinated chickens could potentially serve as a reliable indicator of the types of responses induced by this mRNA vaccine. Moreover, for future studies, enhancing antigen presentation efficiency and prolonging presentation time through mRNA construct modifications and targeted delivery to antigen-presenting cells could be explored to maximize T-cell cytotoxicity.

## Figures and Tables

**Table 1:** Real-time PCR primer sequences for chicken target genes.

Gene	Primer Sequence	Accession Number/ Reference
ß-actin	F:5′-CAACACAGTGCTGTCTGGTGGTA-3′ R: 5′-ATCGTACTCCTGCTTGCTGATCC-3′	X00182
IFN-γ	F: 5′-ACACTGACAAGTCAAAGCCGCACA-3′ R: 5′-AGTCGTTCATCGGGAGCTTGGC-3′	X99774
IFN-α	F: 5′-ATCCTGCTGCTCACGCTCCTTCT-3′ R: 5′-GGTGTTGCTGGTGTCCAGGATG-3′	[22]
IFN-β	F: 5′-GCCTCCAGCTCCTTCAGAATACG-3′ R: 5′-CTGGATCTGGTTGAGGAGGCTGT-3′	[23]
IL-2	F: 5′-TGC AGT GTT ACC TGG GAG AAG TGG T-3′ R: 5′-ACT TCC GGT GTG ATT TAG ACC CGT-3′	NM_204153.1
IL-1β	F: 5′-GTGAGGCTCAACATTGCGCTGTA-3′ R: 5′-TGTCCAGGCGGTAGAAGATGAAG-3′	[24]
MDA-5	F: 5′-GCAAAACCAGCACTGAATGGG-3′ R: 5′-CGTAAATGCTGTTCCACTAACGG-3′	[25]
MyD88	F: 5′-AGCGTGGAGGAGGACTGCAAGAAG-3′ R: 5′-CCGATCAAACACACACAGCTTCAG-3′	[26]
OAS	F:5′-AGAACTGCAGAAGAACTTTGTC-3′ R:5′-GCTTCAACATCTCCTTGTACC-3′	[25]
PKR	F: 5′-TGGTACAGGCGTTGGTAAGAG-3′ R: 5′-GAGCACATCCGCAGGTAGAG-3′	[25]
Mx1	F: 5′-GGACTTCTGCAACGAATTG-3′ F: 5′-TCCCACAAGTTCATCTGTAAG-3′	[24]
IFIT5	F: 5′-CAGAATTTAATGCCGGCTATGC-3′ R: 5′-TGCAAGTAAAGCCAAAAGATAAGTGT-3′	[24]
IRF7	F: 5′-CTCCCCTCCTCCAAAAGCTG-3′ R: 5′-CTGGGAGCGAAGGAGGAATG-3′	[24]
TRIF	F: 5′-GCTGACCAAGAACTTCCTGTGC-3′ R: 5′-AGAGTTCTCATCCA AGGCCACC-3′	[27]
gB	F: 5′-CGGTGGCTTTTCTAGGTTCG-3′ R: 5′-CCAGTGGGTTCAACCGTGA-3′	[28]
pp38	F: 5′-AAGGGTGATGGGAAGGCGATAG-3′ R: 5′-GCATACCGACTTTCGTCAAGATG-3′	[28]

## Data Availability

The original contributions presented in the study are included in the article, further inquiries can be directed to the corresponding author.
